# Ischemia Reperfusion Injury after Gradual versus Rapid Flow Restoration for Middle Cerebral Artery Occlusion Rats

**DOI:** 10.1038/s41598-018-20095-9

**Published:** 2018-01-26

**Authors:** Wan-wan Xu, Ying-ying Zhang, Juan Su, Ao-fei Liu, Kai Wang, Chen Li, Yun-e Liu, Yi-qun Zhang, Jin Lv, Wei-jian Jiang

**Affiliations:** 1 Department of Vascular Neurosurgery, New Era Stroke Care and Research Institute, General Hospital of the PLA Rocket Force, Beijing, 100088 China; 2 Department of Nuclear and Radiation Injury, General Hospital of the PLA Rocket Force, Beijing, 100088 China

## Abstract

Ischemia-reperfusion injury (IRI) is an important cause of adverse prognosis after recanalization in patients with acute occlusion of major intracranial artery (AOMIA). Here, we provided data indicating that gradual flow restoration (GFR) would be superior to rapid flow restoration (RFR) in alleviating cerebral IRIs in middle cerebral artery occlusion (MCAO) rats. A total of 94 MCAO rats with 15, 30 and 60-minute occlusion were randomly assigned to receive either GFR or RFR intervention. There were significant differences between GFR and RFR group in mean neurological severity score (1.02 versus 1.28; p < 0.05), median infarct ratio (0.016 versus 0.12; p < 0.001), median neuronal apoptosis ratio (1.81 versus 14.46; p < 0.001), and mean histopathological abnormality score (0.92 versus 1.66; p < 0.001). In addition, these differences were mainly distributed in 30-minute and 60-minute occlusion rats, not in 15-minute occlusion rats. These results indicated that GFR rather than RFR could effectively alleviate cerebral IRIs in MCAO rats, especially in rats with longer occlusion duration, suggesting that GFR may be particularly applicable to AOMIA patients who are presented to neurointerventionalists in the later-time of recanalization therapy window.

## Introduction

Acute occlusion of the major intracranial artery (AOMIA) is associated with gloomy prognosis for most stroke patients, despite being treated with intravenous r-tPA within 3 or 4.5 hours of the time window^1,2^. Fortunately, recent multiple randomized controlled trials consistently demonstrated that the prognosis gets significantly improved after timely mechanical recanalization therapy with the use of new generations of thrombectomy devices^3–7^. However, the morbidity and mortality were still high in AOMIA patients who underwent thrombectomy treatment, ranging from 29% to 67% at 3 months^8,9^. The poor outcomes may be related to ischemia-reperfusion injury (IRI) and futile reperfusion as well^10,11^. Therefore, it is necessary to seek out the practical and effective strategies for IRI prevention and treatment during AOMIA recanalization.

IRIs are intractable problems characterized by a cascade of deleterious inflammatory responses and cell death. With the understanding of IRIs, the concept of controlled reperfusion was recently proposed, including composition-controlled reperfusion (*i.e*. ionic content, nutrients and acid-base balance) and condition-controlled reperfusion (*i.e*. pressure, flow and temperature)^12,13^. The protective effects of flow- or pressure-controlled reperfusion had been demonstrated by most studies in various organ models, such as myocardium^14^, skeletal muscle^15^, lung and kidney^16,17^. However, there had been limited experimental studies on the condition-controlled reperfusion of brain, as far as we know.

In the present study, we attempt to test the hypothesis that gradual flow restoration (GFR) within certain time window would be superior to rapid flow restoration (RFR) in alleviating cerebral IRI in middle cerebral artery occlusion (MCAO) rats.

## Result

### Relative CBF

**Figure 1:**
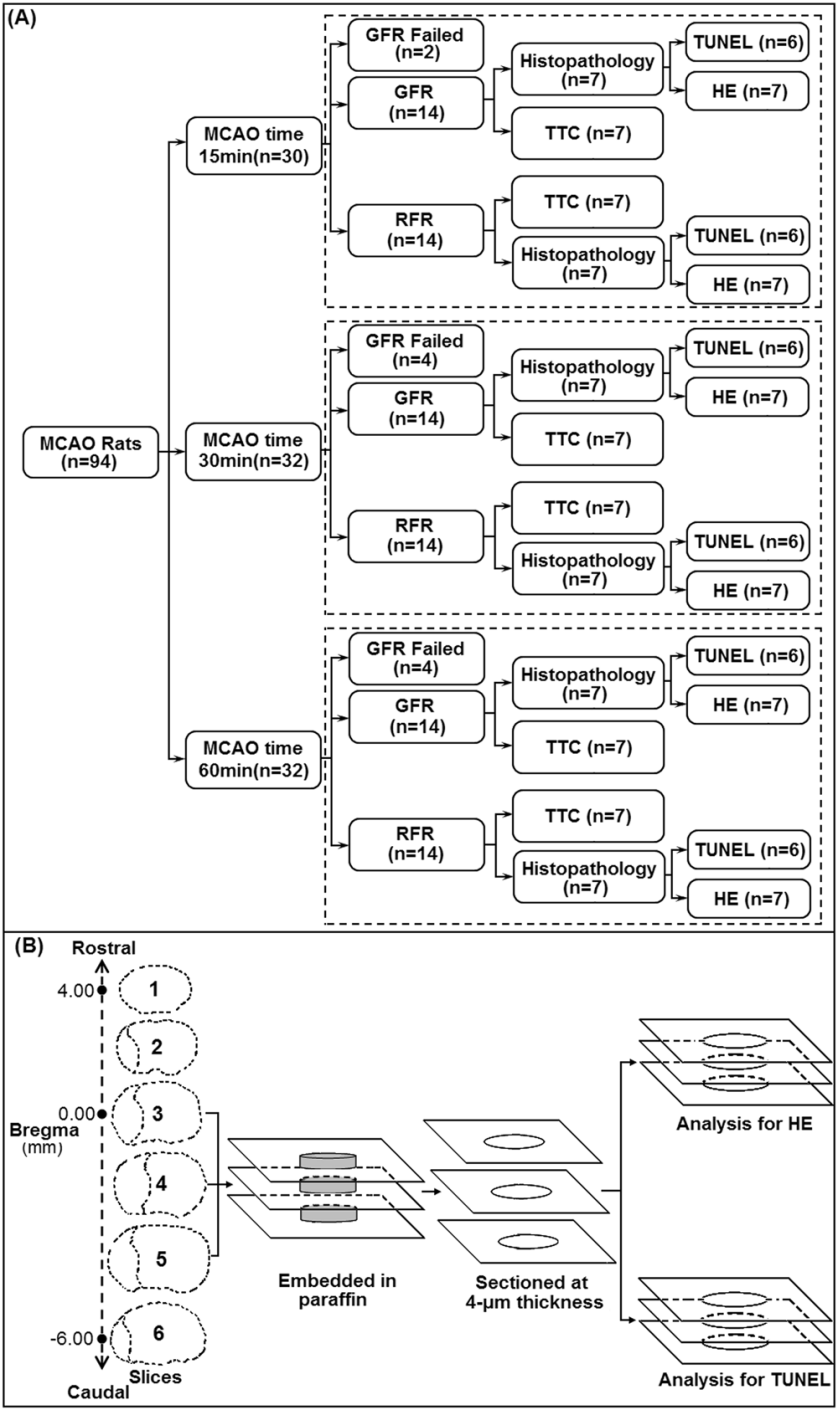
Illustration of experimental group (**A**) and brain tissue sampling procedure of HE and TUNEL analysis (**B**) in MCAO rats treated with GFR and RFR intervention.

A total of 94 MCAO rats were used for this program, and each 42 rats that obtained success of GFR or RFR intervention were enrolled in this study (Fig. 1A). Overall success rates of GFR and RFR intervention were 80.8% (42/52) and 100% (42/42), respectively (*p* < 0.05). The success rates of GFR intervention for 15-, 30- and 60-minute MCAO rats were 87.7% (14/16), 77.8% (14/18) and 77.8% (14/18), respectively.

**Figure 2:**
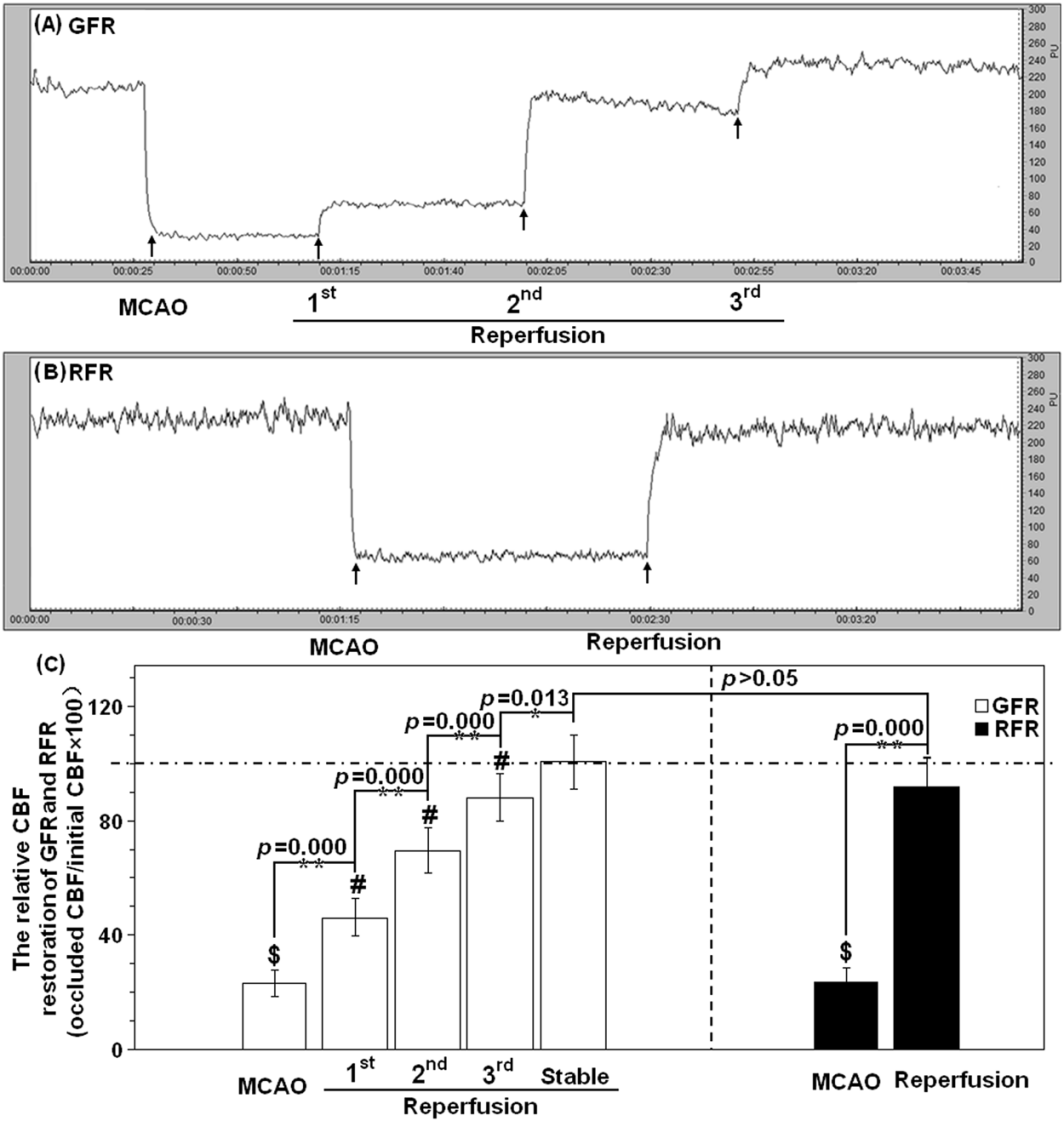
CBF of MCAO rats receiving GFR and RFR by Laser Doppler Flowmetry (LDF). (**A**) Representative LDF of CBF reperfusion in MCAO rats with GFR mode. (**B**) Representative LDF of CBF reperfusion in MCAO rats with RFR mode. (**C**) Comparison of relative alternations in CBF reperfusion undergoing GFR and RFR intervention. The relative CBF data were calibrated by the percentage of pre-occlusion values of CBF and represented as means% ± SD%. The symbol $ means the statistical significance of relative CBF after occlusion versus baseline, # means statistical significance of CBF increment in three-step reperfusion of GFR. *p < 0.05 or **p < 0.01 means statistical significance of relative CBF after reperfusion versus occlusion in each model. The dotted lines mean CBF baseline of rat MCA in GFR and RFR model.

There was no difference in the relative CBF of MCAO rats before GFR and RFR (23.18% ± 1.51% versus 23.56% ± 1.60%, *p* > 0.05) or after GFR and RFR (100.69% ± 2.98% versus 91.65% ± 3.36%, *p* > 0.05), but an obvious distinction in the form of the CBF restoration (Fig. 2). The GFR led to a gradual CBF restoration after 3 times of filament withdrawal with an average increment of 21.67% ± 3.96% for each time, and the RFR caused a sudden CBF restoration.

### Neurological deficit

**Figure 3:**
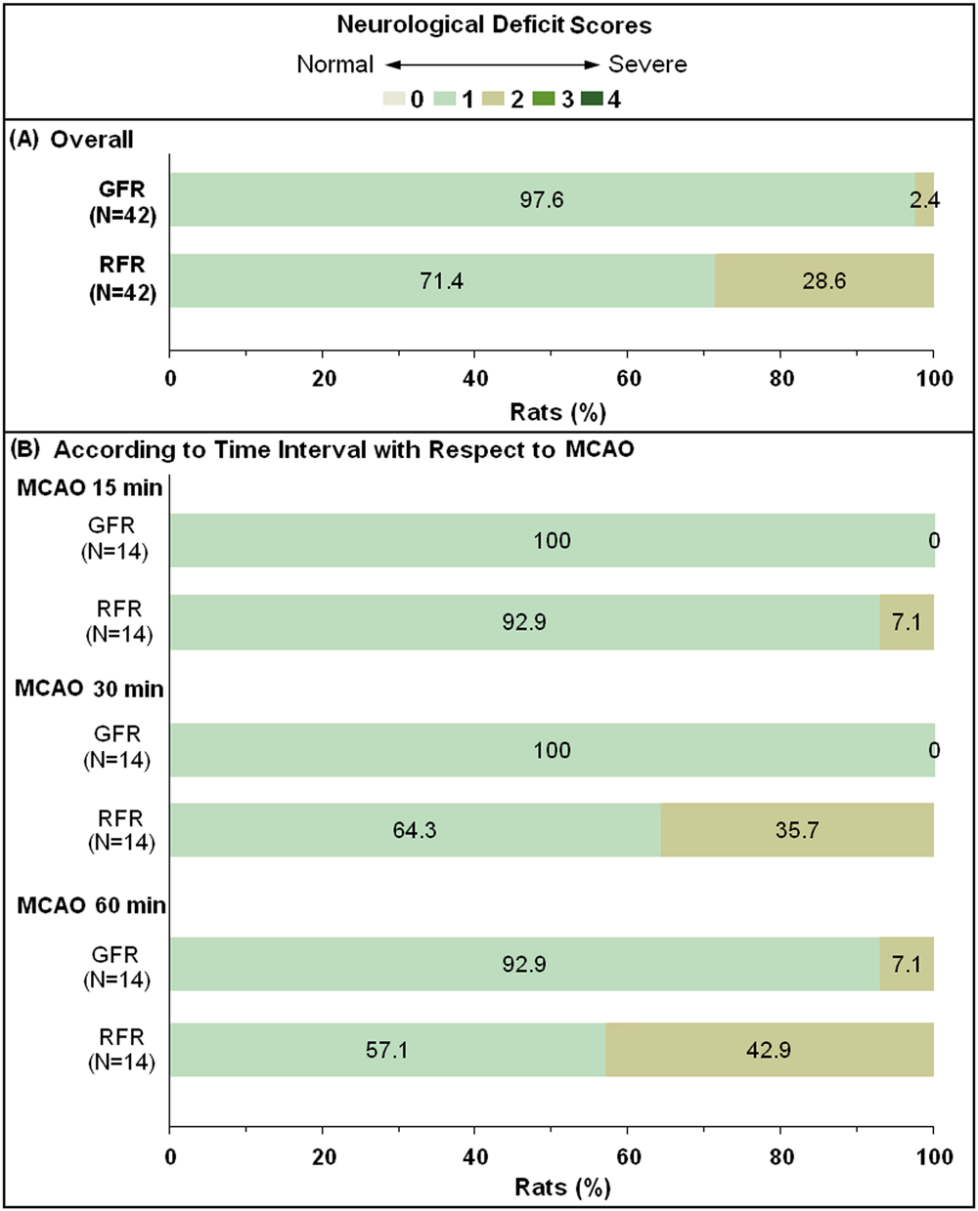
Comparison of neurological deficit of MCAO rats undergoing GFR and RFR intervention. The neurologic deficit scores were evaluated at 24 h after reperfusion with GFR and RFR mode according to a modified Hunter’s method with a scale of 0–4. (**A**) Overall distribution of the neurological deficit scores in GFR and RFR group. (**B**) Distribution of the neurological deficit scores in GFR and RFR group according to time interval with respect to MCAO. Data were presented as a percentage of the number of sample in each score to the total numbers of corresponding samples. Wilcoxon rank-of-rank tests were used to analyze the significant differences between the GFR and RFR groups and there was a shift of the distribution of neurological deficit scores toward mild outcomes in GFR group at 30 or 60 min after MCAO, rather than at 15 min, compared with that in RFR group.

The neurological deficit score at 24 hours was 1.02 (95% CI 1.00–1.08) after GFR, significantly lower than 1.28 (95% CI 1.16–1.43, *p* < 0.05) after RFR. Furthermore, the significant difference existed between GFR and RFR for 30-minute MCAO rats (1.00, 95% CI 1.00–1.00 versus 1.36, 95% CI 1.12–1.62, *p* < 0.05), and 60-minute ones (1.07, 95% CI 1.00–1.23 versus 1.43, 95% CI 1.18–1.70, *p* < 0.05), but did not for 15-minute ones (1.00, 95% CI 1.00–1.00 versus 1.07, 95% CI: 1.00–1.25, p > 0.05) (Fig. 3).

### Infarct size

**Figure 4:**
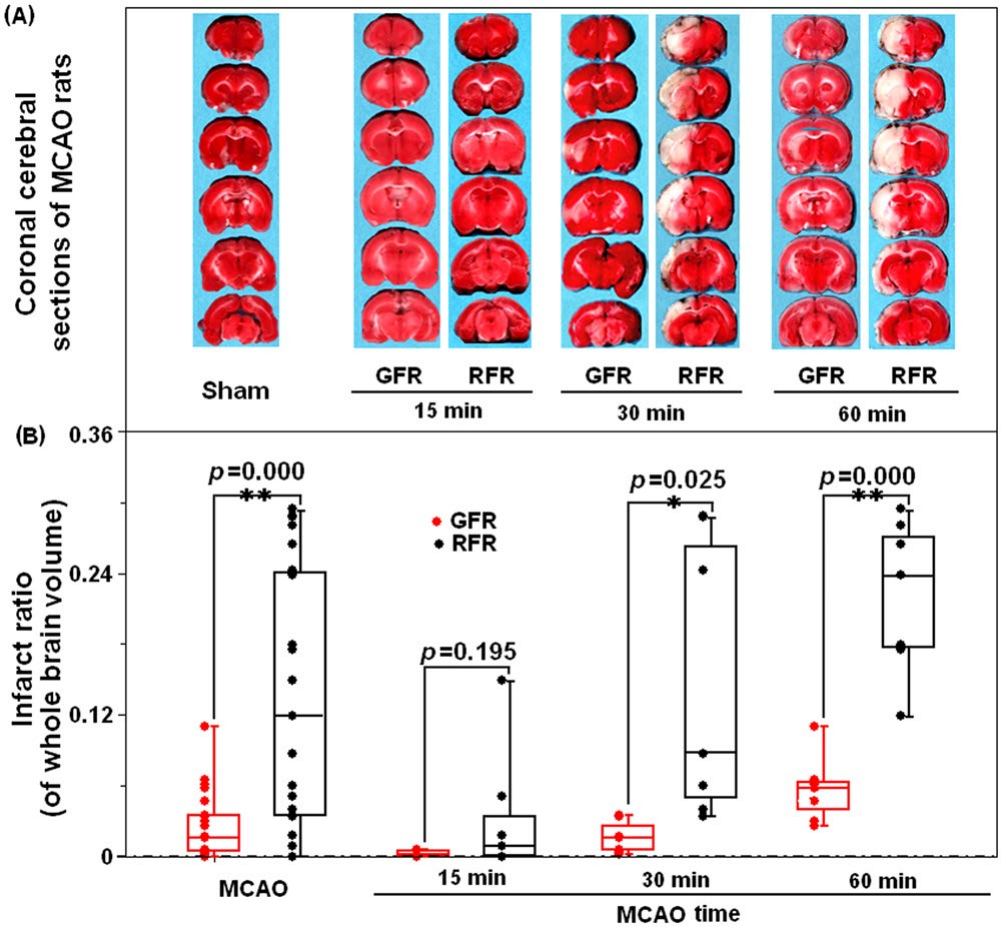
Comparison of ischemic lesion volume of brain in MCAO rats undergoing GFR and RFR intervention. (**A**) Representative TTC stained coronal sections indicating areas of healthy tissue (red) and ischemic injury (white) for each group. Compared with RFR group, the GFR could restrain the brain injury induced by different intervals of MCAO, which was represented as the significantly decreased area of while color. (**B**) Total volumes of ischemic lesion in the ipsilateral hemisphere, expressed as a percentage of the total brain volume, were compared in the GFR and RFR groups after 15-, 30- and 60-minute MCAO treatments. Each value was presented as dot plot of the raw data, overlaid by box and whisker plot (median, first and third percentile, range). Significance was determined by independent Student’s *t* test. *p < 0.05 and **p < 0.01 represent the statistical significance of ischemic lesion volume in GFR-treated group compared with that in RFR-treated group.

The relative infarct size was 0.016 (IQR 0.004–0.035) after GFR, significantly lower than 0.12 (IQR 0.034–0.242, *p* < 0.001) after RFR (Fig. 4). The difference was significant between GFR and RFR for 30-minute MCAO rats (0.016, IQR 0.005–0.0026 versus 0.088, IQR 0.05–0.26, *p* < 0.01), and 60-minute ones (0.058, IQR 0.039–0.063 versus 0.24, IQR 0.18–0.27, *p* < 0.001), but not for 15-minute ones (0.000, IQR 0.000–0.005 versus 0.009, IQR 0.000–0.034, *p* > 0.05), which were consistent with the differences in the neurological deficit (Fig. 3B).

### Histopathological damage

**Figure 5:**
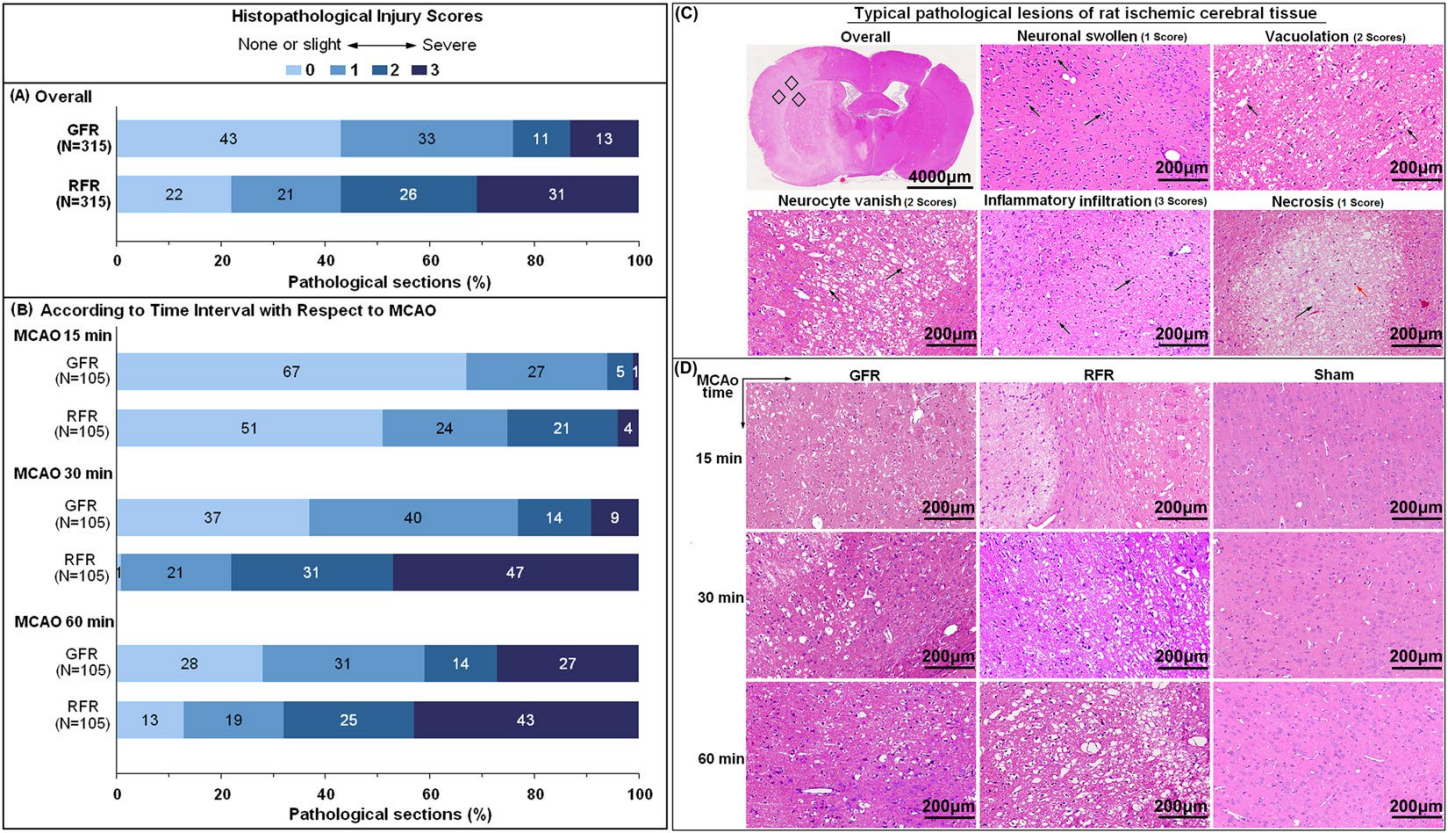
Histopathological injuries of cortex in MCAO rats undergoing GFR and RFR intervention. After assessment of neurological deficit score, brains were removed and fixed in 4% neutral paraformaldehyde. Then, the brain tissues were embedded in paraffin and sectioned at 4 μm thickness in the coronal plane. The sections were stained with haematoxylin and eosin (H&E), and examined under light microscopy (magnification 200×). The histopathological damages were quantified by scoring the extents of the total lesion including focal cerebral edema, vacuolation, neuronal vanish, inflammatory infiltration as well as neuronal necrosis with a scale of 0–3. (**A**) Overall histopathological lesions score in MCAO rats undergoing GFR and RFR. (**B**) Histopathological lesions score according to time interval with respect to MCAO. Data were presented as a percentage of the number of sample in each score to the total numbers of corresponding samples. Significance was determined by Wilcoxon rank-of-rank tests. (**C**) Typical pathological lesions of rat cerebral issue. Red arrow represents photo site of typical inflammatory cell infiltration; black arrows represent each of these typical pathological lesions. Scores of the histopathological lesion are 1 score for neuronal swollen and necrosis, 2 scores for vacoulation and neurocyte vanish, and 3 scores for inflammatory infiltration, respectively. (**D**) Histological changes of cortex in MCAO rats undergoing GFR and RFR. Photomicrographs showed more significant neuronal damages and inflammatory infiltrations in the RFR group than in GFR group, in contrast with no notable morphological changes in sham group. Scale bar = 200 μm.

The mean score of overall abnormality of histopathology in GFR group was 0.92 (95% CI 0.81–1.04) with the dominant score of 0 and 1 point (accounting for over 70%), significantly lower than 1.66 (95% CI 1.53–1.79, *p* < 0.001) in RFR group with the dominant score of 2 and 3 point (accounting for over 50%) (Fig. 5A and B, Supplementary Table 1). The difference was also significant between GFR and RFR for 15-minute, 30-minute, and 60-minute MCAO rats (Supplement Table S1).

### Neuronal apoptosis

**Figure 6:**
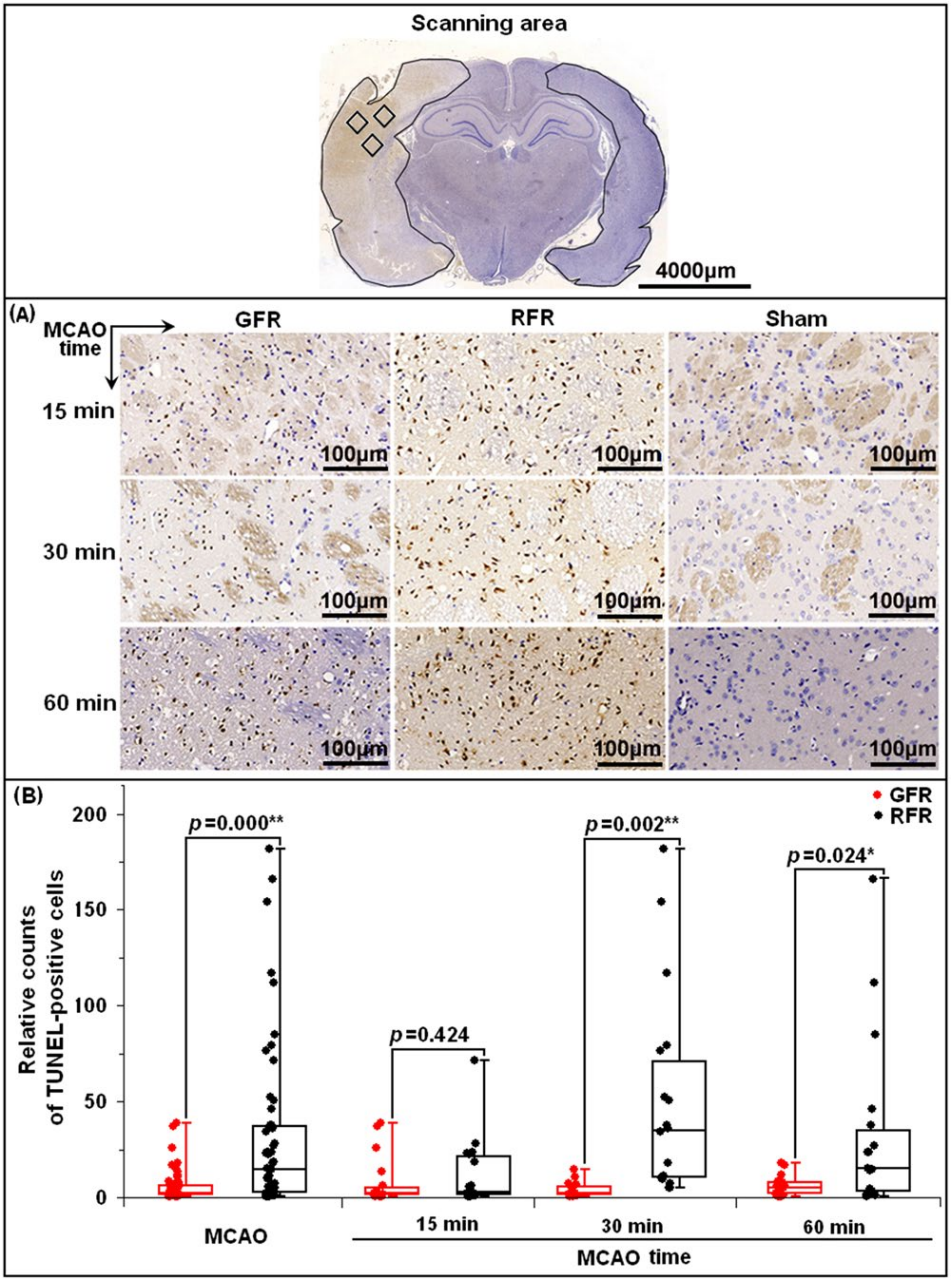
Neuronal apoptosis of ischemic cortex in MCAO rats undergoing GFR and RFR intervention. (**A**) Representative photomicrographs of TUNEL-positive cells (brown staining) in the MCA territory of the ischemic cortex (magnification 400×) after 15, 30 and 60 min MCAO of rats in GFR and RFR intervention. Scale bar = 100 μm. (**B**) Comparison of neuronal apoptosis of rats following GFR and RFR treatment after 15-, 30- and 60-minute MCAO. The TUNEL-positive cells were quantified by NuclearQuant module software (3D, HISTECH Ltd., Budapest, Hungary) and represented as dot plot of the raw data, overlaid by box and whisker plot (median, first and third percentile, range). Significance was determined by independent Student’s *t* test. *p < 0.05 and **p < 0.01 means statistical significance in GFR group versus RFR group.

The relative count of TUNEL-positive cells per unit area in MCAO rats was 1.81 (IQR 1.04–6.43) after GFR, significantly lower than 14.46 (IQR 2.42–37.13, *p* < 0.001) after RFR (Fig. 6). Moreover, the GFR alleviated the apoptosis more significantly than the RFR for 30-minute MCAO rats (1.38, IQR 1.087–5.90 versus 35.01, IQR 10.16–70.64; *p* < 0.01), and for 60-minute ones (5.17, IQR1.59–7.62 versus 15.08, IQR 2.65–34.88, *p* < 0.05), but not for 15-minute ones (1.45, IQR 0.88–5.19 versus 2.24, IQR 1.18–21.47, *p* > 0.05) (Fig. 6).

## Discussion

Despite mounting evidence of the protective effects of controlled reperfusion in some organs, this is the first study showing the effectiveness of flow-controlled reperfusion as a cerebroprotective strategy in focal cerebral infarction. In the present study, we provided data confirming the hypothesis that GFR is superior to RFR in alleviating cerebral IRI, with the evidence of significantly reduced neurological deficit, histopathological damage, infarct size and neuronal apoptosis in MACO rats after GFR intervention. Furthermore, the GFR benefits were demonstrated in 30-minute and 60-minute MCAO rats, but not in 15-minute MCAO rats. The neurological deficit score, infarct size and neuronal apoptosis in 30-minute and 60-minute MCAO rats after GFR were significantly lower than those after RFR. However, there was no difference between the GFR and RFR for the 15-minute MCAO rats in the neurological deficit (1.00, 95% CI 1.00–1.00 versus 1.07, 95% CI: 1.00–1.25, *p* > 0.05), infarct size (0.000 [IQR 0.000–0.005] versus 0.009 [IQR 0.000–0.034], *p* > 0.05) and neuronal apoptosis (1.45 [IQR 0.88–5.19] versus 2.24 [IQR 1.18–21.47], *p* > 0.05).

Generally, the most common durations of MCA occlusion in transient focal cerebral ischemia models are 60-, 90- and 120- minute, with the infarct size ranging from around 20% to 50% of the ipsilateral hemisphere after 24 hours of reperfusion^18,19^. However, human strokes within time window are mostly small in size, ranging from about 4.5% to 14% of the ipsilateral hemisphere^18^. In the present study, we used the 15-, 30-, and 60-minute occlusion time to produce a more diverse infarct volume, with the relative ratio ranging from 0.9% in the 15-minute group, and 8.8% in the 30-minute group to 24% in the 60-minute group (Fig. 4B). We believe our rat model with these occlusion durations can well model the common cases of human stroke within time window and malignant infarction exceeding time window. Using this model, we found GFR could benefit the 30-minute and 60-minute MCAO rats, but not the 15-minute MCAO rats, suggesting that the strategies of endovascular recanalization for AOMIA patients may be time-dependent: GFR for those presented at later time of 6 to 8 hours after stroke onset and RFR for those at earlier time.

In agreement with our findings, Gao and colleague also observed the significant neuroprotective effect of gradual reperfusion in rat CCA/dMCAO model, with the evidence of 33.2% reduction of infarct size after 30-minute of ischemia^20^. However, Allen *et al*.^21^ using a porcine global brain ischemia model demonstrated that low-pressure controlled reperfusion (<50 mmHg), which was protective in other organs, exhibited no beneficial effects on the brain after 30-minute of global cerebral ischemia. Although the protective efficacy of controlled reperfusion had been extensively documented in a variety of experimental settings, such as acute myocardial infarction, ischemic limb and solid organ transplantation, there still had limited experimental studies to clarify the neuroprotective effects of controlled reperfusion on cerebral ischemia^22,23^. Several studies in ischemic myocardium and skeletal muscle attributed the IRIs protection to the effects of low-pressure controlled reperfusion^24,25^ and hypoxemic controlled reperfusion^26^, including reduction of shear stress injury to endothelium, limitation of ROS production and inhibition of inflammatory cells infiltration. In the current study, we found the GFR obviously reduced the inflammatory cells infiltration and neuron apoptosis in 30- and 60-minute MCAO groups. Inflammatory debris is considered as one of the main mechanical components that blocks the capillary bed connecting distal branches of major cerebral artery territories, and then induces neuronal apoptosis in ischemic penumbra^27^. On the other hand, activation of inflammatory reactions can induce the overproduction of ROS, which is needed to further fuel neuronal apoptosis^28^. Thus, our histological results suggested that GFR benefits the cerebral ischemic injury probably through the mechanism of reducing inflammatory cells infiltration and neuron apoptosis in penumbra of MCAO rats.

Additionally, we confirmed that the GFR intervention could be successfully applied on the MCAO rat model, in which the focal CBF of MCA territory could be gradually restored by stepwise withdrawal of occlusion filament, and thus could well simulate the situation of gradual flow restoration during intravascular thrombectomy in AOMIA patients. The success rate of GFR intervention was 80.8% (42/52), slightly lower than the 100% (42/42) of RFR intervention. The primary reason for the failure of GFR in 10 MCAO rats was incorrect CBF increments during gradual reperfusion in this study (7/10) and the failed rats were most commonly found in the 30- and 60-minute groups (8/10). These results had clear clinical relevance because GFR intervention could be easily applied to AOMIA patients during intravascular thrombectomy without any additional equipment and pharmacologic agents.

There were also some limitations in our study. Firstly, we only conducted the short-term experimental observations at 24 hours after reperfusion, therefore, it was not sufficient to evaluate the long-term neuroprotective effect of GFR intervention. In addition, we did not conduct additional functional behavioral analysis such as refined sensorimotor tests and cognitive tests which will help strengthen our understanding of the long-term neuroprotection of GFR. Secondly, this study did not set the permanent occlusion, thus hindering the determination of maximum protective time of GFR. Finally, the underlying mechanisms of the neuroprotective effects of GFR intervention, as well as more detailed animal behavior experiments, should be further studied in the future.

In conclusion, our study demonstrated that the newly established GFR intervention, rather than the conventional RFR intervention, had a more potent neuroprotective effect on transient ischemic injury in MCAO rats model. Therefore, application of GFR intervention coupled with mechanical thrombectomy could be effective on preventing cerebral IRIs and improving outcomes of AOMIA patients, which should be studied in the future.

## Methods

### Animals and ethical approval

Male Sprague Dawley rats (3–8 weeks old, weighing 240–260 g) were purchased from the Vital River Laboratory Animal Technology Company, Beijing, China. Animals were maintained at 20–25 °C with a 12 h light–dark cycle and allowed food and water *ad libitum* except under certain experimental conditions. All the animal researches were approved and conducted in facilities with programs accredited by The Ethics Committee of PLA Rocket Force General Hospital (NO. 2014013), and were carried out in strict accordance with the recommendations in the Guide for Care and Use of Laboratory Animals of the National Institutes of Health.

### Establishment of MCA occlusion model

The rats were anesthetized with 10% chloral hydrate (300 mg/kg) via intraperitoneal injection. Middle cerebral artery occlusion (MCAO) model was then created, as described by Longa *et al*. with slight modification^29,30^. Briefly, right common, external and internal carotid artery (CCA, ECA and ICA) were exposed, and dissected away from adjacent nerves. After ligation of the distal trunk of ECA, and electrocoagulation of the branches at the proximal trunk of ECA, the CCA was temporarily clamped with microvascular clips. Then, an ECA incision was made, and a nylon monofilament of 0.26 mm in diameter with a silicone tip of 0.34 mm in diameter (Jialing L3600, Guangzhou, China) was gently introduced via the incision and advanced into the distal ICA to occlude the MCA. Successful establishment of MCAO was defined as a reduction of cerebral blood flow (CBF) in the MCA territory to less than 30% of baseline CBF.

Eight sham MCAO rats underwent all the surgical procedures except for the monofilament advancing into the distal ICA to occlude the MCA. These rats were used for the TTC staining controls.

### Measurement of cerebral blood flow

CBF in the MCA territory was monitored and measured by Laser Doppler Flowmetry (LDF, moorVMS-LDF Monitor, England). The laser Doppler probe was positioned over the thinned skull approximately 5 mm lateral and 3 mm caudal to bregma overlying the MCA territory. CBF data were continuously collected with dedicated software and CBF photographs were captured automatically. Relative CBF after surgery was a ratio of CBF after occlusion or recanalization to baseline CBF before occlusion.

### Randomization protocol

The MCAO rats with different occlusion durations (15-, 30- and 60- minute, n = 28) were randomized into GFR and RFR groups using the random number table generated by the RAND () function of Microsoft Excel package. If the random number can be divisible by 2, the rats are allocated to GFR group (remainder = 0); otherwise, the rats are allocated to RFR group (remainder = 1). If the number of rats assigned to each group is not equal, the extra rats in the larger number group are randomly selected out and reassigned to another group. If the rats failed to GFR or RFR, additional MCAO rats are recruited to meet the pre-designed numbers of each group.

### GFR or RFR for MCAO rats

This study planned to enroll each 42 MCAO rats that obtained successful GFR and RFR intervention, respectively. Each intervention group consisted of respective 14 rats that experienced 15-, 30- and 60-minute occlusion of MCA (Fig. 1A). A three-step filament withdrawal was used in GFR intervention: step 1, clamped contralateral ICA with microvascular clip to avoid compensating blood from the contralateral ICA via azygous ACA, then slowly pulled out the occlusion filament until CBF increase to about 40–49% of baseline CBF, which was maintained for 2 minutes; step 2, mildly withdrew the filament until CBF increase to about 60–69% of baseline CBF for 2 minutes; step 3, withdrew the filament out and took off the contralateral temporary clip. The successful GFR intervention was defined as: (1) a three-phase gradual CBF recovery to baseline within 5 minutes, and (2) the flow gains in each phase is about 20–30% of the baseline, and (3) each phase of stepwise CBF lasts about 1 minute. The failure of GFR was defined as any one of the followings: (1) CBF did not restore to 80% of the baseline after the filament was completely withdrew out; (2) CBF restoration did not follow a three-phase gradual increasing manner; or (3) CBF increment for each phase is less than 10% or greater than 50% of the baseline value. Rapid withdrawal of occlusion filament was used in RFR intervention. The success of RFR intervention was defined as a quick CBF recovery to 80% of baseline within 30 seconds. If not, it was defined as a failure. After surgical procedures, rats were recovered in the homeothermic temperature system (TR-200, Fine Science Tools Inc., Foster City, CA) to maintain body temperature between 36.5–37.5 °C.

### Assessment of neurological deficits

Neurological severity scoring of the enrolled rats at 24 hours of post-recanalization was independently assessed by a researcher who did not know recanalization modality, using a 5-point scale: 0 point, normal with no observable neurologic deficit; 1 point, mild deficit with flexion of the contralateral torso and the forelimb upon lifting of the animal by its tail; 2 points, moderate deficit, circling to the contralateral side but normal posture at rest; 3 points, severe deficit with leaning to the contralateral side at rest; 4 points, very severe deficit with no spontaneous motor activity.

### Measurement of infarct size

A half of the enrolled GFR and RFR rats with 15-, 30-, and 60-minute MCAO were used for this purpose. They were anesthetized after evaluation of neurological deficit, and then sacrificed. Their brains were dissected rapidly and frozen in −20 °C for 20 min. The whole brains were then cut into 6 coronal sections with 2-mm thickness and stained with 2% TTC (2,3,5-Triphenylterazolium chloride, Sigma-Aldrich, United Kingdom) at 37 °C for 20 min, followed by overnight postfixation in 4% neutral paraformaldehyde solution. Unstained area was defined as infarct lesion and demarcated for analysis by Image J software (NIH Image, Version 1.61, Bethesda, USA). Overall infarct volume was summarized by each infarct volume in 6 sections, which was corrected by overall infarct volume × (1 − [ipsilateral hemisphere volume − contralateral hemisphere volume]/contralateral hemisphere volume) for compensating edema effect. Infarct size was expressed as a ratio of corrected overall infarct volume to whole brain volume.

### Histological analysis and TUNEL assay

Another half of the enrolled rats were used. They were anesthetized with chloral hydrate after assessment of neurological deficit, and perfused with saline, followed with 4% neutral paraformaldehyde. Brains were removed and fixed in 4% neutral paraformaldehyde, and then, cut into 6 sections at 2-mm thickness along coronal plane from baseline of cerebral peduncle upward. The third to fifth slices were respectively embedded in paraffin. Then, 2 thin-slices of 4-μm thickness were made from each slice per the standard procedure (Fig. 1B). After being mounted on polylysine-coated slides, and air dried, one thin-slice of each slice was stained for hematoxylin and eosin (H&E); and another one for terminal deoxynucleotidyl transferase (TdT)-mediated dUTP-biotin nick end labeling (TUNEL), which was cover slipped using DPX mounting media and left to dry for 2 days before image capture.

The HE-stained thin-slices were used to assess overall abnormality of histopathology, including neuronal swollen, vacuolation, neurocyte vanish, inflammatory infiltration as well as neuronal necrosis. The abnormality, independently assessed by a pathologist, was scored 0 scale, no or few abnormality in ipsilateral hemisphere; 1 score, moderate abnormality accounting for 1/3 to 1/2 ipsilateral hemisphere; 2 scales, severe abnormality for 1/2 to 2/3 one; and 3 scales, very severe abnormality for >2/3 one.

The TUNEL assay thin-slices were used to detect the apoptotic cells, which are brown staining within their nuclei (TUNEL-positive ones). The thin-slices were treated according to the instruction of cell death detection kit (Roche, Basel, Switzerland), and scanned by Pannoramic 250 slide scanner (3D, HISTECH Ltd., Budapest, Hungary). Then, the TUNEL data were collected by NuclearQuant module software (3D, HISTECH Ltd., Budapest, Hungary) and represented as relative counts of TUNEL-positive cells per unit area, *ie*., a ratio of TUNEL-positive cell percentage of ipsilateral to contralateral hemisphere.

### Statistical analysis

The SPSS Statistics software package (v19.0) was used for the manipulation and statistical analysis of data. Relative CBF of GFR and RFR group was expressed as mean ±SD, and compared with one-way analysis of variance (ANOVA) for each time withdrawal of GFR and independent Student’s *t* test for blocking and final recanalization between GFR and RFR. Scores of neurological severity and histopathological abnormality were presented as mean and 95% confidence interval (CI), and compared with Wilcoxon rank-of-rank tests. Ratios of infarct size and neuronal apoptosis were expressed by median and interquartile range (IQR), and compared with 2-independent samples nonparametric tests. A *p* value < 0.05 with two-side was considered statistically significant.

## Electronic supplementary material

Supplementary material
