# Supplementary material for: Ischemia Reperfusion Injury after Gradual versus Rapid Flow Restoration for Middle Cerebral Artery Occlusion Rats
Source: Sci Rep. 2018 Jan 26;8:1638. doi: 10.1038/s41598-018-20095-9 (PMC5786000; doi:10.1038/s41598-018-20095-9)
Supplement: Supplementary file 1 — Supplementary material [file 41598_2018_20095_MOESM1_ESM.pdf]

Ischemia Reperfusion Injury after Gradual versus Rapid Flow Restoration for Middle Cerebral Artery Occlusion Rats

Wan-wan Xu (MD)[1#], Ying-ying Zhang (PhD)[1#], Juan Su (MD)[1], Ao-fei Liu (MD)[1], Kai Wang (MD)[1], Chen Li (MD)[1], Yun-e Liu (MD)[1], Yi-qun Zhang (MD)[1], Jin Lv (PhD) [1, 2*], Wei-jian Jiang (MD, PhD)[1*]

ONLINE SUPPLEMENT

Supplement table S1 Histopathological scores of brain damages in MCAO rats undergoing GFR and RFR intervention.

Pathological changes	MCAO		MCAO interval (min)					
			15		30		60	
	GFR	RFR	GFR	RFR	GFR	RFR	GFR	RFR
Overall	0.92(0.81-1.04)**	1.66(1.53-1.79)**	0.40(0.28-0.52)**	0.77(0.60-0.95)**	0.97(0.79-1.17))**	2.24(2.08-2.39)**	1.4(1.17-1.61)**	1.97(1.76-2.18)*
NS	1.00(0.85-1.17)**	1.37(1.22-1.53)**	1.10(0.81-1.44)**	1.76(1.46-2.07)**	0.95(0.69-1.26)	1.14(0.95-1.35)	0.95(0.72-1.23)	1.19(0.95-1.42)
V	1.35(1.03-1.65)**	2.05(1.71-2.33)**	0.43(0.17-0.74)	0.81(0.40-1.24)	1.48(1.00-2.00)**	2.90(2.75-3.00)**	2.14(1.65-2.63)	2.43(1.94-2.86)
NV	0.75(0.48-1.00)**	1.51(1.21-1.78)**	0.10(0.00-0.24)	0.33(0.10-0.58)	0.71(0.35-1.15)**	2.29(2.00-2.56)**	1.43(0.94-1.95)	1.90(1.41-2.38)
II	1.14(0.90-1.40)**	1.90(1.59-2.17)**	0.33(0.13-0.56)	0.76(0.38-1.13)	1.24(0.88-1.60)**	2.62(2.35-2.84)**	1.86(1.39-2.33)	2.33(1.82-2.79)
N	0.38(0.22-0.55)**	1.48(1.16-1.74)**	0.05(0.00-0.16)	0.19(0.04-0.36)	0.48(0.22-0.78)**	2.24(1.92-2.53)**	0.62(0.32-0.93)**	2.00(1.57-2.43)*

After assessment of neurological deficit score, 7 rats were used for histological examination in each group and the histopathological damages were quantified by scoring the extents of neuronal swollen (NS), vacuolization (V), neuronal vanish (NV), inflammatory infiltration (II) as well as necrosis (N) of neurons with a scale of 0−3. Data were expressed as mean, 95% CI. Significance was determined by Wilcoxon rank-of-rank

tests. Asterisks represent a significant difference between GFR group and RFR group (*p<0.05, and ** p<0.01).

Supplement table S2 Body weights in MCAO rats undergoing GFR and RFR intervention.

MCAO groups	Body weights (g)			
	15min (n=28)	30min (n=28)	60min (n=28)	MCAO Total (n=84)
GFR	251.00±4.55	249.16±4.44	249.94±4.12	250.04±4.51
RFR	248.55±4.03	249.55±5.90	249.78±4.99	249.26±4.74
***P* value**	*0.767*	*0.336*	*0.563*	*0.711*
